# Perceived Healthcare Discrimination and Barriers to Care for Older Adults Living with Traumatic Brain Injury

**DOI:** 10.1093/geroni/igaf122.3823

**Published:** 2025-12-31

**Authors:** Yalian Pei, Haowei Wang

**Affiliations:** Syracuse University, Syracuse, New York, United States; Syracuse University, Syracuse, New York, United States

## Abstract

Traumatic brain injury (TBI) is a leading cause of morbidity and mortality in the aging population. Older adults living with TBI have higher risks of hospitalization and often need frequent healthcare. Despite these risks, limited research has examined the quality of healthcare and barriers to healthcare access among older adults living with TBI. Drawing on a large, nationally representative dataset from the All of Us Research Program, this study examined perceived discrimination in healthcare settings and its associations with healthcare utilization and delayed care among older adults with and without TBI. In the sample of N = 70,070 older adults aged 60 and above, 1,713 had a clinical diagnosis of TBI from Electronic Health Records. Respondents were evaluated on a 7-item scale of perceived healthcare discrimination and reported their healthcare utilization and delayed care in the last year. After adjusting for sociodemographic and health conditions, regression results show that compared to those who did not have TBI, older adults living with TBI were more likely to utilize healthcare (OR = 1.29, p< .05) but were not more likely to delay care (OR = 1.01, p=.883). Among older adults with TBI, perceived discrimination in healthcare settings was significantly associated with higher risks of delaying care (OR = 1.43, p<.001) but was not significantly associated with healthcare utilization (OR = 0.96, p=.227). These findings underscore the need to improve healthcare quality and reduce discriminatory experiences among older adults living with TBI.

